# Application of ^1^H-MRS in end-stage renal disease with depression

**DOI:** 10.1186/s12882-020-01863-0

**Published:** 2020-06-15

**Authors:** Jiachen Wang, Tong Zhou, Jihua Liu, Jingjun Shangguan, Xuejun Liu, Zhiming Li, Xiaoming Zhou, Yande Ren, Chengjian Wang

**Affiliations:** 1 Department of Medical Imaging Center, The Affiliated Hospital of Qingdao University, 16 Jiang-Su Road, Qingdao, PR China; 2 Department of Radiology, Qilu Hospital of Shandong University, Qingdao Branch, 758 He-Fei Road, Qingdao, PR China

**Keywords:** End-stage renal disease, Depression, Proton magnetic resonance spectroscopy, Frontal lobe

## Abstract

**Background:**

To investigate the metabolite changes in the frontal lobe of the end-stage renal disease (ESRD) patients with depression using proton magnetic resonance spectroscopy (^1^H-MRS).

**Methods:**

All subjects were divided into three groups: ESRD patients with depression (30 cases), ESRD patients without depression (27 cases) and 32 normal subjects. ESRD with depression patients were further divided into two groups according to the severity of depression: 14 cases of ESRD with severe depression group (Hamilton Depression Rating Scale (HAMD) score ≥ 35) and 16 cases of ESRD with mild to moderate depression group (20 ≤ HAMD score<35). ^1^H-MRS was used in brain regions of all subjects to measure N-acetylaspartate/creatine (NAA/Cr), choline-containing compounds/creatine (Cho/Cr) and myo-inositol/creatine (MI/Cr) ratios of the frontal lobe. Correlations between the metabolite ratio and HAMD score as well as clinical finding were confirmed, respectively.

**Results:**

ESRD patients with depression showed lower NAA/Cr ratio and higher Cho/Cr ratio compared with ESRD patients without depression and normal subjects. NAA/Cr ratio was negatively correlated with the HAMD score. Cho/Cr ratio was positively correlated with the HAMD score. There were positive correlations between NAA/Cr ratio and blood urea notrogen (BUN) as well as creatinine (CRE) concentration, respectively. There was a negative correlation between Cho/Cr ratio and sodium concentration. The Cho/Cr ratio was positively correlated with the potassium concentration.

**Conclusions:**

MR spectroscopy identified some metabolite changes in ESRD patients with depression.

## Background

End-stage renal disease (ESRD), also called kidney failure, is the later stage of chronic kidney disease (CKD) in which the kidneys cease function on a permanent basis [1]. The only treatment options for kidney failure are regular course of dialysis or a kidney transplant. ESRD patients are confronted with long-term treatment, heavy ideological burden, strong economic pressure and other problems, which can put the patients under a very stressful condition. Such psychological stress contributes greatly to the induction of various psychological disorders, especially depression [2]. Studies have shown that more than 80% of ESRD patients had psychological disorders [3]. Depression is the most prevalent comorbid psychiatric condition with a prevalence rate as high as 20 to 25% among ESRD patients [4]. Depression can accompany ESRD patients from the onset and progression of the disease. This negative emotion will seriously affect the physical and mental health and lower the quality of life of patients. What’s worse, it will also increase the mortality rate and suicide rate [5]. Therefore, it is essential for clinical ESRD patients to establish a good internal defense mechanism and improve the early diagnosis and treatment of depression.

Currently the diagnosis of ESRD with depression is mainly based on clinical findings and Hamilton Depression Rating Scale (HAMD), which are very subjective with low detection rate. Thus, the objective and quantitative biomarkers for early diagnosis are urgently needed. Proton magnetic resonance spectroscopy (^1^H-MRS) is a noninvasive technique with high spatial resolution, which can provide a quantitative analysis of biochemical composition in the living human brain. Different molecules have unique magnetic resonance spectra, which can be quantified by taking the area under the signal curve and measuring it against the curve of a standard metabolite [6–8]. ^1^H-MRS can reliably detect metabolites such as N-acetylaspartate (NAA), choline-containing compounds (Cho), creatine (Cr) and myo-inositol (MI) in the brain. NAA level represents the number and integrity of neurons in the detection area. Cho level mainly reflects the change of cell membrane synthesis and degradation. Cr level shows the energy metabolism. Most studies used Cr as a reference for other metabolites due to the Cr concentration in physiological and pathological conditions maintained in a relatively steady state [9].

^1^H-MRS has been widely used to study depression and depression-related diseases. The metabolite ratios obtained with MRS may be useful markers for early diagnosis of ESRD with depression. Patients with depression usually exhibited decreased NAA/Cr ratio, increased Cho/Cr ratio and elevated MI/Cr ratio in frontal lobe, hippocampus and other brain regions compared with normal subjects [10–13]. The recent researches have found that ^1^H-MRS is valuable for the diagnosis and evaluation of post stroke depression, Parkinson’s disease with depression and bipolar depression [14–17].

Previous studies have indicated that the frontal lobe and hippocampus in brain regions are closely associated with depression [11, 18]. The frontal lobe is the largest sector of the hemisphere, and often it is claimed that it has developed more than other areas in humans [19]. The prefrontal cortex is close to basal ganglia region, thalamus and other cerebral cortexes through commissural and projection fibers. It is the most important area that affects neural and spiritual activities and helps regulate the emotional activities of human beings. So it is defined as the pathophysiological region of emotional abnormality, which plays an important role in emotional regulation and information transmission [20]. Due to the small volume of hippocampus and its close distance to the sulcus, it is difficult to locate and quantify the regions of interest (ROIs) using single voxel ^1^H-MRS. Therefore, the hippocampus was not included in the detection range of ^1^H-MRS in this experiment.

In this prospective study, we divided all subjects into 3 groups: ESRD with depression, ESRD without depression and normal subjects. We evaluated the metabolite changes in the brain using ^1^H-MRS. The correlations between the metabolite ratio and HAMD score as well as clinical presentation were confirmed. The clinical significance is that provides theoretical basis for early diagnosis and assessment of depression severity of ESRD patients.

## Methods

### Participants

This study was conducted from January 2016 to March 2017 at the Affiliated Hospital of Qingdao University (Qingdao, China), which is a tertiary hospital with over 5000 beds. The study was approved by the Institutional Review Board and was compliant with the Health Insurance Portability and Accountability Act (HIPAA). Informed consent was obtained from all patients prior to enrollment in the study.

The inclusion criteria for this study were that all patients (a) met the diagnosis of ESRD with glomerular filtration rate (GFR) less than 15 ml/min per 1.73 m^2^, (b) didn’t undergo or stop hemodialysis for at least 2 weeks, the type of the hemodialysis was conventional hemodialysis and the patients were dialyzed thrice-weekly with a Fresenius 4008 dialysis machine (Fresenius Medical Care, Bad Homburg, Germany) using Fresenius Polysulfone®-based dialysis membrane (Fresenius S.E., Bad-Homburg, Germany), and (c) evaluated with HAMD (HAMD score ≥ 20, patients will be recruited into ESRD with depression group. Particularly, ESRD with severe depression when HAMD score ≥ 35, ESRD with mild to moderate depression when 20 ≤ HAMD score < 35; HAMD score < 8, patients will be recruited into ESRD without depression group). The subjects who met any of the following criteria were excluded: (a) significant illness such as hypertention, cardiac or liver diseases, (b) central nervous system diseases such as cerebral vascular disease, infection, neoplasm or neurodegenerative disease, (c) primary depression, schizophrenia or other psychologic disease, (d) alcohol or drug use and (e) not cooperative. We randomly selected 156 patients from all of the hospital’s approximately 550 patients with ESRD. Among excluded subjects, 73 patients or family did not give consent and 26 did not meet other eligibility criteria such as illness. The remaining 57 subjects composed the study sample. 30 patients were enrolled into ESRD with depression group, including 14 patients with severe depression and 16 patients with mild to moderate depression. 27 patients were enrolled into ESRD without depression group. Another 32 age- and gender- matched healthy subjects were recruited from local community as control group. All healthy subjects’ HAMD score < 8. Cranial MRI scans revealed no abnormal findings for all healthy subjects.

All subjects took routine blood test right after MRI examination. ESRD patients took additional blood biochemistry tests, including the blood urea notrogen (BUN) and creatinine (CRE) levels and blood sodium and potassium concentrations. The MRI and tests were performed on the same day for each patient, who didn’t undergo hemodialysis treatment or stop hemodialysis for at least 2 weeks. The demographics and clinical data of healthy controls and ESRD patients are summarized in Table 1.

**Table 1:** Baseline characteristics and clinical data of the healthy controls and ESRD patients with/without depression (mean ± standard deviation)

Characteristics	ESRD patients with depression (*n* = 30)	ESRD patients without depression (*n* = 27)	Healthy controls (*n* = 32)
Age (years)	33.4 ± 8.6	36.6 ± 9.3	36 ± 7.2
Gender (M/F)	19/11	17/10	20/12
Cause of ESRD			
Diabetes mellitus, n (%)	7/30 (23.3)	5/27 (18.5)	/
Hypertension, n (%)	4/30 (13.3)	3/27 (11.1)	/
Course of disease (months)	47.2 ± 14.1	45.3 ± 13.7	/
Dialysis time (months)	20.6 ± 5.5	18.2 ± 6.7	/
HAMD score	27.1 ± 2.16	6.24 ± 1.88	5.42 ± 1.87
Hemoglobin (g/dL)	11.0 ± 3.7	10.3 ± 1.9	12.6 ± 1.7
Glucose (mmol/L)	6.79 ± 2.56	6.31 ± 2.04	5.2 ± 0.68
eGFR (mL/min/1.73 m^2^)	5.72 ± 2.68	6.94 ± 2.81	/
K_t_/V	1.7 ± 0.6	1.6 ± 0.3	/

### Imaging acquisition

**Fig. 1:**
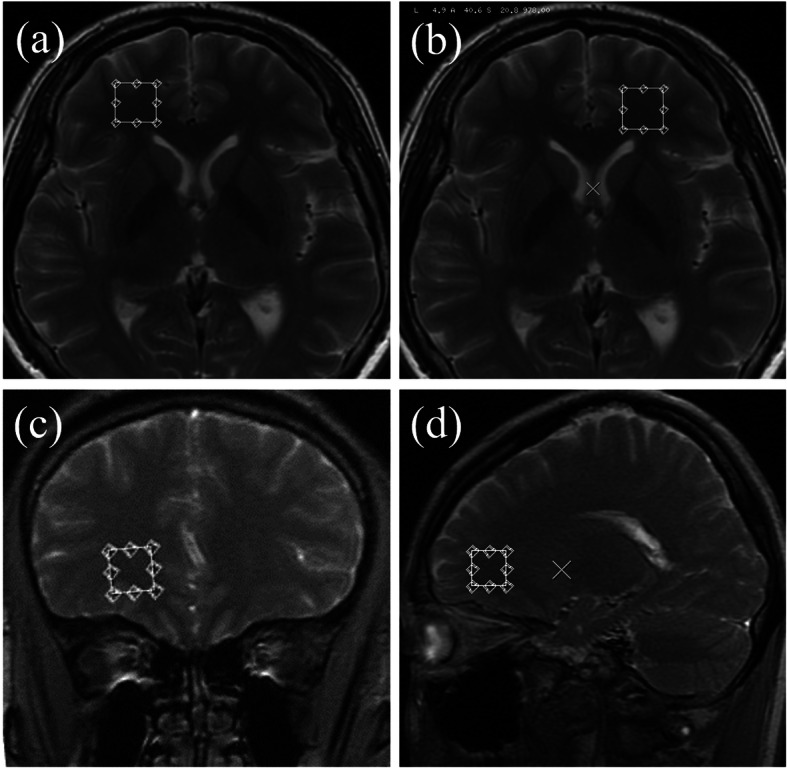
The ROI location maps of **a**, **b** axial, **c** coronary and **d** sagittal

MRI was performed on a GE Signa 3.0 T whole-body scanner equipped with phased-array head coil (32-channel). Imaging protocol included axial T1-weighted image (TR/TE = 2500/24), T2-weighted image (TR/TE = 4020/120) and axial FLAIR image (TR/TE/TI = 8000/165/2100, 5 mm slice thickness). Single voxel spectroscopy (SVS) was performed by using a spin-echo sequence (point resolved spectroscopy) with water suppression by mean of selective excitation. The regions of interest (ROIs) were placed in bilateral frontal lobe close to the frontal horn of lateral ventricle, avoiding cerebral spinal fluid (CSF) (Fig. 1). Sequence parameters included the following: TR/TE = 1500/35 ms, NEX =8, voxel size 15 × 15 × 15 mm.

Chemical shift imaging (CSI) spectrum was used for semi-quantitative analysis of the detected substance. The Fourier transformed data set was phase corrected (zero and first order), followed by baseline correction. Metabolite areas for NAA, Cr, Cho and MI in the frontal lobe were determined by using a frequency domain line-fitting program. The mean NAA/Cr, Cho/Cr and MI/Cr ratios were calculated.

### Statistical analysis

An unpaired student t test was used to test the difference between groups of ESRD with depression, ESPR without depression and normal subjects. A *p*-value of less than 0.05 was considered significant. Pearson correlations were performed between metabolite ratios, HAMD score and lab exams. All data analysis was conducted using SPSS 22.0.

## Results

### Comparison of general information

Table 1 shows the baseline characteristics and clinical data of the subjects in this study. The age and gender were not significantly different between the patients with ESRD and healthy controls. The ESRD patients had much higher glucose than healthy subjects. There was no significant difference in cause of ESRD, course of disease, dialysis time and HAMD score between ESRD patients with depression and ESRD without depression.

### Comparison between ESRD with depression and ESRD without depression

Table 2 displays NAA/Cr ratios of ESRD with depression group were significantly lower than that of ESRD without depression. Whereas Cho/Cr ratios of ESRD with depression group were significantly higher compared with the group of ESRD without depression. There were no significant differences of MI/Cr ratios between ESRD patients with depression and without depression.

**Table 2:** Metabolite ratios between ESRD with depression and without depression (mean ± standard deviation)

Metabolite ratios		ESRD with depression	ESRD without depression	*P* values
NAA/Cr	right	1.63 ± 0.16	1.72 ± 0.12	0.013
	left	1.60 ± 0.19	1.71 ± 0.19	0.015
Cho/Cr	right	1.24 ± 0.12	1.10 ± 0.17	0.036
	left	1.20 ± 0.12	1.08 ± 0.13	0.012
MI/Cr	right	0.74 ± 0.10	0.73 ± 0.13	0.145
	left	0.70 ± 0.10	0.69 ± 0.11	0.087

### Comparison between ESRD with depression and normal subjects

Table 3 shows NAA/Cr ratios were significantly lower in ESRD with depression group compared with normal subjects. Whereas Cho/Cr and MI/Cr ratios were significantly higher in ESRD with depression group compared with normal subjects.

**Table 3:** Metabolite ratios between ESRD with depression and normal subjects (mean ± standard deviation)

Metabolite ratios		ESRD with depression	Normal subjects	*P* values
NAA/Cr	right	1.63 ± 0.16	1.74 ± 0.18	0.011
	left	1.60 ± 0.19	1.76 ± 0.16	0.008
Cho/Cr	right	1.24 ± 0.12	1.00 ± 0.12	0.005
	left	1.20 ± 0.12	1.02 ± 0.14	0.003
MI/Cr	right	0.74 ± 0.10	0.64 ± 0.14	0.025
	left	0.70 ± 0.10	0.61 ± 0.13	0.031

### Comparison between ESRD without depression and normal subjects

Only MI/Cr ratios showed significant difference between ESRD patients without depression and normal subjects (Table 4). There were no significant differences in other metabolite ratios.

**Table 4:** Metabolite ratios between ESRD without depression and normal subjects (mean ± standard deviation)

Metabolite ratios		ESRD without depression	Normal subjects	*P* values
NAA/Cr	right	1.72 ± 0.12	1.74 ± 0.18	0.064
	left	1.71 ± 0.19	1.76 ± 0.16	0.061
Cho/Cr	right	1.10 ± 0.17	1.00 ± 0.12	0.057
	left	1.08 ± 0.13	1.02 ± 0.14	0.072
MI/Cr	right	0.73 ± 0.13	0.64 ± 0.14	0.027
	left	0.69 ± 0.11	0.61 ± 0.13	0.036

### Comparison between ESRD with severe depression and ESRD with mild to moderate depression

Table 5 exhibited NAA/Cr ratios were lower in ESRD with severe depression group compared with ESRD patients with mild to moderate depression. Cho/Cr ratios were higher in ESRD with severe depression group compared with ESRD with mild to moderate depression subject. There were no significant differences of MI/Cr ratios between the two groups.

**Table 5:** Metabolite ratios between ESRD with severe depression and ESRD with mild to moderate depression (mean ± standard deviation)

Metabolite ratios		ESRD with severe depression	ESRD with mild to moderate depression	*P* values
NAA/Cr	right	1.59 ± 0.20	1.66 ± 0.19	0.012
	left	1.54 ± 0.22	1.66 ± 0.23	0.008
Cho/Cr	right	1.28 ± 0.16	1.19 ± 0.15	0.019
	left	1.30 ± 0.18	1.22 ± 0.16	0.010
MI/Cr	right	0.76 ± 0.13	0.73 ± 0.15	0.028
	left	0.70 ± 0.14	0.71 ± 0.13	0.021

### The ^1^H-MRS spectra in the bilateral frontal lobes

**Fig. 2:**
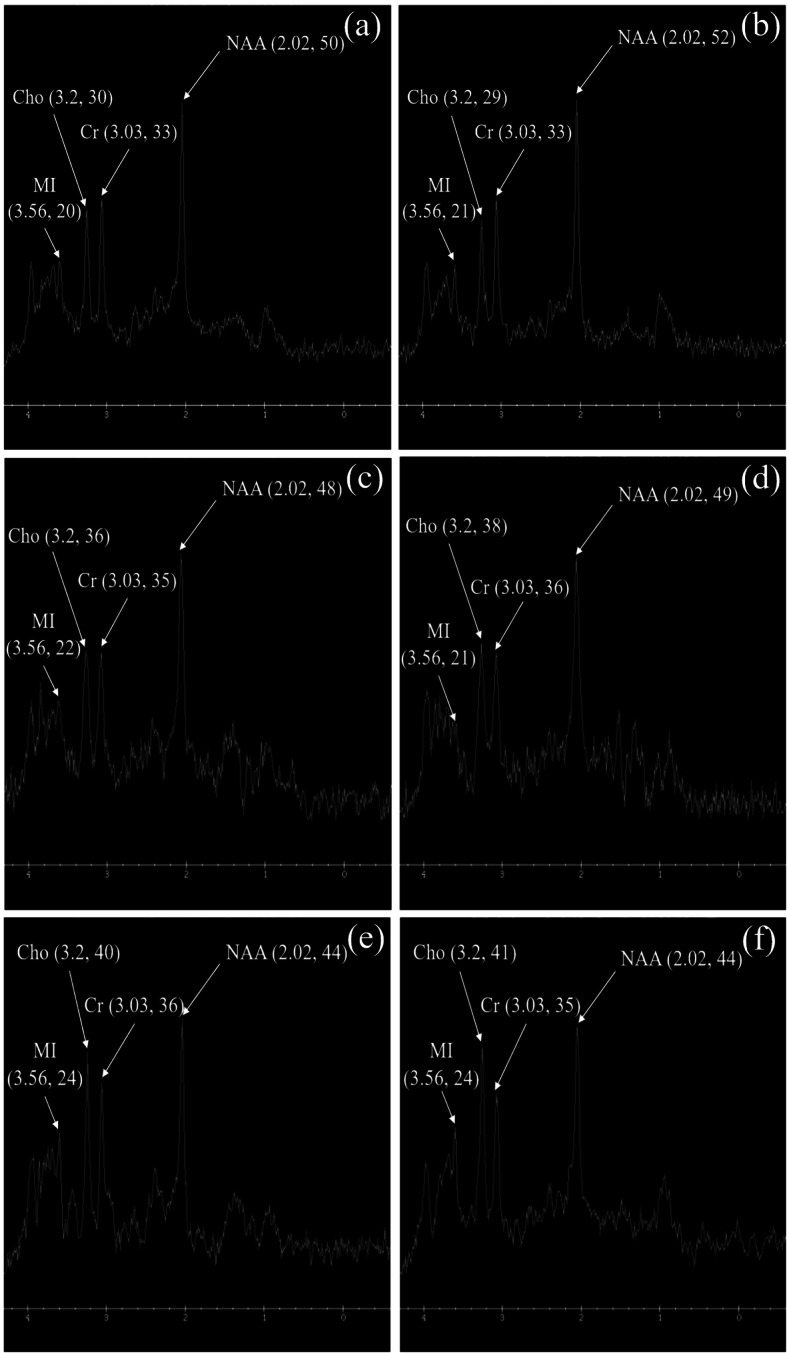
The ^1^H-MRS spectra in the bilateral frontal lobes of **a**, **b** normal control, **c**, **d** ESRD without and **e**, **f** with depression

As shown in Fig. 2a, b, the frontal lobe spectra of the two sides were basically similar. In the ESRD without depression group (Fig. 2c, d), there was a slight decrease in the amplitude of NAA in bilateral frontal lobes, a slight increase in Cho amplitude, and little change in the amplitudes of MI and Cr compared with the normal control group. Compared with the ESRD without depression group and the normal control group, the NAA amplitude of bilateral frontal lobes in ESRD with depression group was significantly reduced, the Cho and MI amplitudes were significantly increased, and Cr amplitude was not significantly changed (Fig. 2e, f).

### Correlation between metabolite ratios and clinical findings

**Fig. 3:**
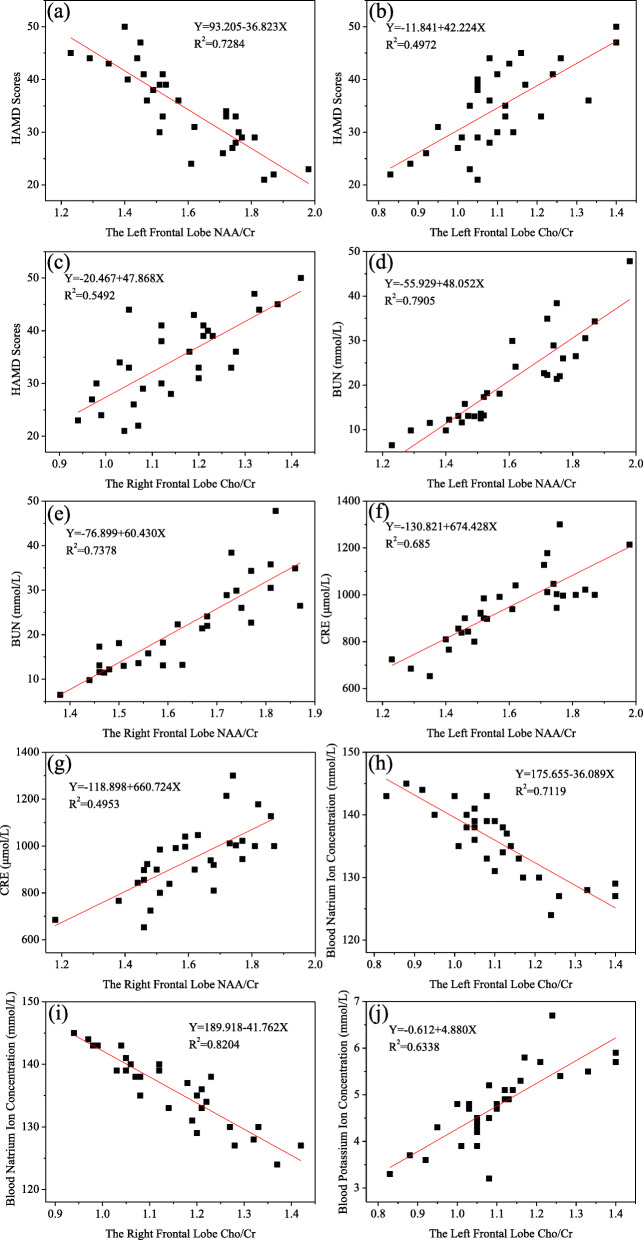
The correlation diagrams of **a** NAA/Cr ratios and HAMD scores in the left frontal lobe, **b**, **c** Cho/Cr ratios and HAMD scores in the left and right frontal lobes, **d**, **e** NAA/Cr ratios and BUN level in the left and right frontal lobes, **f**, **g** NAA/Cr ratios and CRE level in the left and right frontal lobes, **h**, **i** Cho/Cr ratios and blood sodium concentration in the left and right frontal lobes and **j** Cho/Cr ratios and blood potassium concentration in the right frontal lobe of ESRD patients with depression

There was a significant negative correlation between NAA/Cr ratios and HAMD scores in the left frontal lobe of ESRD with depression group (Fig. 3a). There was a significant positive correlation between Cho/Cr ratios and HAMD scores in the bilateral frontal lobes of ESRD with depression group (Fig. 3b, c). As we can see from Fig. 3d, e, f, g, there were significant positive correlations between NAA/Cr ratios and BUN as well as CRE levels in the bilateral frontal lobes of ESRD with depression group, respectively. There was significant negative correlation between Cho/Cr ratios and blood sodium concentration in the bilateral frontal lobes of ESRD with depression group (Fig. 3h, i). There was a significant positive correlation bewteen Cho/Cr ratios and blood potassium concentration in the left frontal lobes of ESRD with depression group (Fig. 3j).

## Discussion

Our study demonstrated that ESRD patients with depression had decreased NAA and increased Cho amplitudes in the frontal lobe compared with ESRD patients without depression and normal subjects. There were significant correlations between NAA/Cr ratios, Cho/Cr ratios, HAMD scores and blood electrolyte levels. Our study indicated that ^1^H-MRS can detect metabolite changes in the brain of ESRD patients with depression.

The mechanisms of depression in patients with ESRD are very complicated but may be related to both psychological and physical abnormalities. Due to kidney dysfunction in patients with ESRD, small molecules such as BUN and other mediums or large molecular substances are retained in the body, leading to metabolic disorders, affecting the metabolism of normal cells and resulting in cell damage and brain dysfunction [21]. On the other side, patients with ESRD are challenged by many stressors, including inability to function well, huge medical cost, failure to maintain their occupation and decreased mobility [22]. The prevalence of depression in patients with ESRD is 3–4 and 2–3 times higher than that in the general population and individuals with other chronic illnesses, respectively. The lifetime risk of depression is estimated to be 5–10% in the general population, the more severe the depression, the higher the risk of mortality [23–25]. Therefore, early diagnosis and treatment of depression in patients with ESRD are very important.

MRS has been used in the diagnosis of depression [26]. NAA functions as an acetyl donor for acetyl coenzyme A and takes part in the synthesis of lipid, including myelin. NAA is a putative neuronal marker because it is localized only in neurons [27]. Hence, the reduction in NAA levels is indicative of neuronal loss and dysfunction. As patients with ESRD become more aggravated, BUN, Cr and other toxins continue to aggregate in the body. The enzyme system that maintains metabolic activity of brain cell is inhibited, including the catalysis synthesis of NAA with aspartic acid N-acetyltransferase [28]. Therefore, abnormalities occur in myelination, which affecting the normal function of nerve conduction and neural network and leading to the development of depression. In addition, the ESRD patients may be accompanied by ischemia and hypoxia of brain cell due to the effects of water-sodium retention and vasoactive substances, which affecting the function of mitochondria and resulting in reduced NAA levels [12, 29].

We found a significantly increased Cho signal amplitude in ESRD patients with depression, which was consistent with previous reports [30–32]. Cho is the precursor of neurotransmitters such as acetylcholine, membrane lipids, phosphatidylcholine and sphingomyelin, and is a marker for the state of membrane phospholipid metabolism [33]. In the brain, the MRS visible Cho resonance primarily arises from phosphocholine and glycerophosphocholine, whereas much smaller contribution from free choline and acetylcholine [34]. Therefore, the elevated Cho signal amplitude most likely reflects high turnover of membrane and damage of myelin or neurons. The Cho signal measures not just Cho but also the underlying phosphocholine and glycerophosphocholine [34, 35]. Elevated Cho level observed in our study may be due to electrolyte imbalance and reduced osmotic pressure in ESRD patients. When the brain cells are subjected to severe hypoxia, the cell membrane will be damaged. Elevated Cho level indicated cell membrane dysfunction of bilateral prefrontal neurons occurred in ESRD patients with depression.

MI is a naturally occurring glucose isomer which has a number of known roles in the brain [36]. It is traditionally considered as a glial marker because it is actively transported into astrocytes and functions in osmoregulation in glial cells of brain. Higher levels probably reflect gliosis [37]. Our study showed that ESRD patients had higher MI/Cr ratios in bilateral prefrontal cortexes compared to normal subjects. But there was no significant difference in the MI/Cr ratios between ESRD patients with and without depression, suggesting that elevated MI/Cr ratio may be due to the metabolism of the ESRD disease itself instead of psychological reason.

The NAA/Cr ratios in the bilateral prefrontal cortexes were positively correlated with the concentrations of BUN and CRE, indicating that the accumulation of toxins such as BUN and CRE may play an important role to reduce NAA level. Our study also showed that there were negative correlation between Cho/Cr ratio and sodium concentration and positive correlation between Cho/Cr ratio and potassium concentration. Hyponatremia and hyperpotassium can cause cytotoxic edema, damage cell membrane and result in elevated Cho level.

Only the changes of ^1^H-MRS metabolites in bilateral frontal lobes were studied in this experiment, while the occurrence of ESRD combined with depression may be related to biochemical pathological changes in multiple brain regions, so the conclusions obtained have certain limitations. Furthermore, the durations of disease and dialysis of ESRD patients are varied, and the effects of different treatment regimens on the metabolism may affect the accuracy of laboratory examination indicators of patients. Also the sample is relatively small and maybe it’s better to present the results as preliminary data. Future study with large sample size will be warranted.

## Conclusions

MR spectroscopy identified some metabolite changes in ESRD patients with depression, which exhibited reduced NAA/Cr and increased Cho/Cr ratios in the frontal lobe.

## Abbreviations

ESRD: End-stage renal disease
^1^H-MRS: Proton magnetic resonance spectroscopy
HAMD: Hamilton Depression Rating Scale
NAA: N-acetylaspartate
Cr: Reatine
Cho: Choline-containing compounds
MI: Myo-inositol
BUN: Blood urea notrogen
CRE: Creatinine
ROI: Regions of interest
HIPAA: Health Insurance Portability and Accountability Act
GFR: Glomerular filtration rate
SVS: Single voxel spectroscopy
CSF: Cerebral spinal fluid
CSI: Chemical shift imaging

## Availability of data and materials

The datasets analysed during the current study are available from the corresponding author on reasonable request.
